# Compression of Cognitive Decline and Cognitive Resilience in Extreme Longevity

**DOI:** 10.1093/geroni/igaf122.4284

**Published:** 2025-12-31

**Authors:** Wenxin Zhang, Wenjie Cai, Albert Hofman, Anand Viswanathan, Susanne van Veluw, Deborah Blacker, Sudeshna Das, Yuan Ma

**Affiliations:** Harvard School of Public Health, Boston, Massachusetts, United States; Johns Hopkins University School of Public Health, Baltimore, Maryland, United States; Harvard School of Public Health, Boston, Massachusetts, United States; Massachusetts General Hospital, Boston, Massachusetts, United States; Massachusetts General Hospital, Boston, Massachusetts, United States; Massachusetts General Hospital, Boston, Massachusetts, United States; Massachusetts General Hospital, Harvard Medical School, Boston, Massachusetts, United States; Harvard School of Public Health, Boston, Massachusetts, United States

## Abstract

Compressing the duration of cognitive impairment is critical to preserve quality of life until the end. To what extent cognitive decline is compressed and cognitive resilience increases with extreme longevity is not well understood. We used data of 13,999 deceased participants from the National Alzheimer’s Coordinating Center cohort, including 8,146 with neuropathological data. Cognitive function was assessed annually (median follow-up: 4.9 years). We evaluated cognitive trajectories before death and cognitive resilience (defined as high neuropathological burden without dementia) across lifespan groups (ages 50-100+). Participants with longer lifespans, particularly centenarians, exhibited slower cognitive decline and shorter periods of cognitive impairment before death, although distinct cognitive trajectories existed among centenarians. Cognitive resilience also increased with longer lifespans, but associated factors varied. APOE ε2 was associated with higher cognitive resilience only in centenarians. Our findings support a general compression of cognitive decline and increased cognitive resilience in extreme longevity.

